# Clinico-Radiological Correlation of Weber’s Syndrome

**DOI:** 10.7759/cureus.51624

**Published:** 2024-01-03

**Authors:** Anand Hatgaonkar, Kajal Hatgoankar, Meet Jobanputra, Prasad Desale

**Affiliations:** 1 Radiodiagnosis, Datta Meghe Medical College, Datta Meghe Institute of Higher Education and Research, Nagpur, IND; 2 Pathology, Datta Meghe Medical College, Datta Meghe Institute of Higher Education and Research, Nagpur, IND; 3 Radiodiagnosis, Jawaharlal Neharu Medical College, Datta Meghe Institute of Higher Education and Research, Wardha, IND

**Keywords:** brain stem stroke, mri brain, weber’s syndrome, oculomotor nucleus, paramedian mid brain infarct

## Abstract

Weber's syndrome, named after Hermann Weber, is characterized by midbrain lesions often caused by strokes, resulting in ipsilateral third nerve palsy, including ptosis and pupillary abnormalities, and contralateral hemiplegia. We discuss a case of a 35-year-old lady with cognitive impairment, right hemiparesis, diplopia, left eye ptosis, and lateral eye deviation. MRI of the brain with contrast suggested an acute infarct in the left-sided paramedian region of the midbrain. The oculomotor nucleus and cerebral peduncle were both affected by an abrupt left-sided paramedian midbrain stroke. The participation of particular midbrain nuclei together with symptoms including drooping eyelids, diplopia, and limb paralysis suggested Weber’s syndrome. An MRI study of the brain is the modality of choice in suspected stroke cases and is more sensitive when it comes to the brainstem lesions. A comprehensive neurological examination with a clinical diagnosis of Weber’s syndrome before radiological investigations is of great help for localizing brain stem lesions and thus aids in early diagnosis and treatment.

## Introduction

Weber’s syndrome is named after the German physician Hermann Weber, who first described it in 1863 [1]. Weber's syndrome is a part of rare clinical syndromes known as crossed paralyses or alternating hemiplegia in which there are ipsilateral motor deficits of cranial nerves and contralateral hemiparesis. Clinical knowledge about the involvement of specific cranial nerves and contralateral hemiparesis is very helpful in localizing the lesion to a specific part of the brainstem [2]. Weber’s syndrome is characterized by a variety of symptoms brought on by a midbrain lesion, usually due to stroke. It entails ipsilateral third nerve palsy, including ptosis, pupillary dilation, and accommodation reflexes, which are present on the same side, and contralateral hemiplegia [3]. If the third nerve nucleus is only partially damaged, the pupil may not be involved [4].

## Case presentation

A 35-year-old woman presented to the emergency department with complaints of cognitive impairment, right-sided hemiparesis, diplopia, left eyelid drooping and eye deviation laterally on the left side. The patient demonstrated minor disorientation upon evaluation. Although there was oculomotor palsy, both pupils were equal and light-reactive.

**Figure 1:**
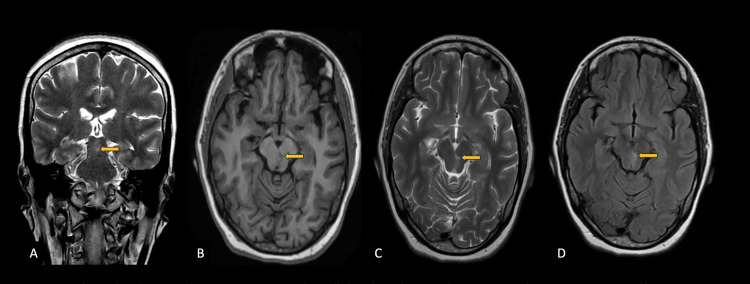
MRI Brain; T1, T2 and FLAIR axial images MRI brain; T1, T2 and fluid-attenuated inversion recovery (FLAIR) images reveal a focal altered signal intensity lesion in the left paramedian region of the midbrain (yellow arrow) appearing hypointense on T1 weighted image (WI) axial plane (B), hyperintense on T2WI in coronal (A); axial (C) and FLAIR axial plane (D).

With the aforementioned complaints, the patient was advised an MRI of the brain with contrast for further evaluation. The MRI study of the brain with contrast revealed an ill-defined altered signal intensity lesion in the left-sided paramedian region of the midbrain, appearing hypointense on T1 weighted images (WIs) and hyperintense on T2WIs and fluid-attenuated inversion recovery (FLAIR) images (Figure 1).

**Figure 2:**
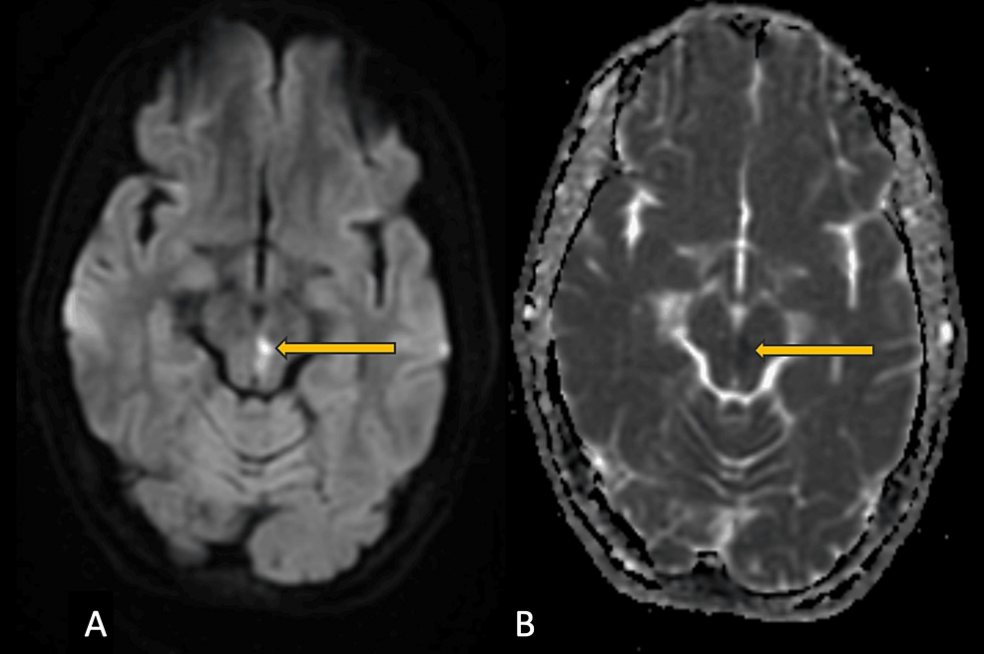
MRI Brain; DWI and ADC images MRI Brain; Diffusion weighted (DW) and apparent diffusion coefficient (ADC) images in the axial plane showing restricted diffusion (yellow arrow) appearing bright on DW image  (A) and corresponding loss of signals on ADC image (B).

It shows restricted diffusion on diffusion-weighted images (DWIs) appearing hyperintense on DWI and hypointense on apparent diffusion coefficient (ADC) images ( Figure 2).

No evidence of blooming was noted on the gradient echo (GRE) suggesting the absence of calcification or haemorrhage. No post-contrast enhancement was noted. MRI brain angiography of the patient did not reveal any abnormality. Imaging features of non-enhancing altered signal intensity lesion with restricted diffusion as described above was suggestive of acute ischemic stroke.

This patient was treated with an IV injection of alteplase (recombinant tissue plasminogen activator (rtPA)), which is the gold standard treatment for ischemic stroke. Due to prompt diagnosis with an MRI brain and timely interventions, the patient attended full recovery with a reversal of symptoms in a few days.

## Discussion

Weber's syndrome is characterized by paramedian midbrain lesions with symptoms of eyelid drooping, diplopia, crossed impairments, either sensory or motor, vertical gaze palsy, and sporadically occurring ataxia [3,4]. 

Every patient with a suspected stroke should have a comprehensive neurologic examination before being further assessed using radiological techniques like CT and MRI [5]. MRI is a very sensitive investigation for evaluating strokes. It can reveal even very small lesions involving the paramedian midbrain and cerebral peduncle, which can cause abrupt midbrain stroke. These small lesions are easily overlooked in the absence of proper clinical evaluation and diagnosis.

In our case, imaging findings were correlative and suggested an acute infarct in the left-sided paramedian region of the midbrain. The oculomotor nucleus and cerebral peduncle were both affected by a left-sided paramedian midbrain stroke [6]. The participation of particular midbrain nuclei together with symptoms including drooping eyelids, diplopia, and limb paralysis confirmed the clinical scenario suggesting Weber’s Syndrome. 

Additionally, it may involve the medial longitudinal fasciculus, the rostral interstitial nucleus of the midbrain, which is dorsal to the red nucleus, resulting in vertical gaze palsy [7]. According to the rostrocaudal theory, the pupillary component, extraocular movement, and elevation of the eyelids make up the fascicular arrangement of the midbrain oculomotor nerve [8].

Hydrocephalus and tonsillar herniation may develop due to mass effect. The imaging findings of extensive posterior circulation infarction and tonsillar herniation, both provide a significant mortality risk. Other syndromes with midbrain stroke are Benedikt, Claude, Parinaud, and Nothnagel syndromes as per the involvement of the different midbrain regions [2,9].

## Conclusions

MRI is the modality of choice in suspected cases of Weber’s syndrome and is a very sensitive investigation in evaluating small brainstem lesions. Comprehensive neurological examination with clinical diagnosis before radiological assessment is of great help in localizing even small brain stem lesions. Clinico-radiological correlation can precisely pinpoint the anatomical location in Weber’s syndrome and thus aids in early diagnosis and treatment.
